# Increasing educational inequalities in self-rated health in Brazil, 1998-2013

**DOI:** 10.1371/journal.pone.0196494

**Published:** 2018-04-30

**Authors:** Flavia Cristina Drumond Andrade, Jeenal Deepak Mehta

**Affiliations:** Department of Kinesiology and Community Health, University of Illinois at Urbana-Champaign, Champaign, IL, United States of America; Yokohama City University, JAPAN

## Abstract

The objectives of this study are to analyze the associations between educational levels and poor self-rated health (SRH) among adults in Brazil and to assess trends in the prevalence of poor self-rated health across educational groups between 1998 and 2013. Individual-level data came from the 1998, 2003 and 2008 Brazilian National Household Survey and the 2013 National Health Survey. We estimate prevalence rates of poor SRH by education. Using multivariable regressions, we assess the associations between educational levels and poor self-rated health. We use these regressions to predict the estimated ratios between the prevalence rates of those in low vs. high education in order to assess if relative changes in poor SRH have narrowed over time. Finally, we tested for statistically significant time trends in adult chronic disease inequalities by education. Results indicate a clear educational gradient in poor SRH. Prevalence ratios show that Brazilian adults with no education have levels of poor SRH that are 7 to 9 times higher than those with some college or more. The difference between those with lowest and highest education increased from 1998 to 2013. Compared to those with no education, there were increases in the prevalence of poor SRH among those with primary and secondary incomplete as well as among those with secondary complete in 2008 and 2013. In conclusion, there is a positive association between poor SRH and low education. Brazil has many social and geographic inequalities in health. Even though educational levels are increasing, there is no improvement in the general subjective health of Brazilians. Health inequalities by race and region highlight the need to improve the health of socially disadvantaged groups in Brazil. Addressing chronic conditions and mental health is needed to improve self-perceptions of health in Brazil as well.

## Introduction

Brazil is marked by health disparities with individuals of lower socioeconomic conditions often exhibiting worse health outcomes across several health outcomes, such as infant mortality, life expectancy and disability [1–4]. These health differences are evident despite the fact that Brazil has been expanding access to health care services following the 1988 constitution, which declared health to be a right of all citizens [5]. The Unified Health System is the major employer of health care professionals in Brazil [5, 6]. However, adequate care is not universal and there are inequalities in health care access across social groups and across geographic areas [6].

Even though disparities in health care access exist, there are other major social health determinants of health, such as education, income, wealth and region that help explain these disparities in Brazil. In the past decades, Brazil has been experiencing major changes in social determinants of health, particularly in educational levels. Since the 1990s, basic education expanded in Brazil. At the same time, the number of students in higher education has doubled [7]. The Gini educational coefficient reduced from 0.48 in 1990 to 0.35 in 2004 [8]. Even though inequalities in education remain, increased access to higher education has mostly benefited Whites and those from higher income households [7]. Therefore, large educational inequalities remain. These changes in social determinants of health, particularly education, along with increase access to health care have the potential to influence health indicators [5], particularly the perception of general health.

The perception of general health, captured by self-rated health, has been shown to be a predictor of chronic disease, disability and mortality [9, 10]. In fact, poor self-rated health is not only a good predictor of mortality, but its predictive effect is comparable to objectively measuring health [11]. In Brazil, poor self-rated health has been shown to be associated with a host of low socioeconomic indicators. In particular, poor self-rated health has been shown to be associated with lower educational levels among adults [12–15]. Higher prevalence of fair and poor health have been found in neighborhoods with lower income and educational levels [16] and among adults with higher material hardship, working load and in lower status occupations [13, 15]. Residing in households with at least one worker unemployed also increased the odds of having poor self-rated health [14]. Nonetheless, previous studies examining socioeconomic differences in self-rated health in Brazil have focused on cross sectional data [12, 13, 16–20], with few using nationally representative data [14, 15].

Even though there are many dimensions of health inequalities in self-rated health in Brazil, socioeconomic differences in health in Brazil are largely driven by differences in educational attainment [21]. Given the recent changes in educational achievement, we believe that educational policies are powerful ways in addressing health inequalities. To our knowledge, no previous study has examined trends in poor self-rated health by educational groups. This study addresses this gap. We used three waves (1998, 2003, and 2008) of the Brazilian National Household Survey (Pesquisa Nacional por Amostra de Domicílios, PNAD) and the 2013 National Health Survey (Pesquisa Nacional de Saúde, PNS) to address two objectives: first, to analyze the associations between educational levels and poor self-rated health and second, to assess trends in the prevalence of poor self-rated health across educational groups between 1998 and 2013. We provide absolute and relative measures of educational inequalities in poor self-rated health following recent studies in the field [22].

## Materials and methods

### Survey and setting

Individual-level data from the 1998, 2003, and 2008 PNAD as well as the 2013 PNS were used in the analyses. PNAD is a repeated cross-sectional in-person household survey that gathers information on sociodemographic traits like education, earnings, and employment of the Brazilian individuals. The PNAD data from 1998, 2003, and 2008 included many questions on health conditions like disease diagnoses, self-rated health, and health insurance. The PNAD incorporated a multistage, probability sampling design in order to provide national estimates related to Brazil’s population. Municipalities were selected at random during the first stage. From each selected municipality, in the second stage, census tracts were randomly selected and the inclusion probability was proportional to the number of households in a census tract. The third stage included randomly selected households for interviews from each chosen census tract.

The PNS is a household-based survey that collects information on the use of health services, health insurance, lifestyle, and health status of people in Brazil. Moreover, the PNS includes data on sociodemographic features like educational attainment. The PNS incorporated a multistage, probability sampling design to provide estimates in regards to Brazil’s population. The PNS sample is also a subsample of the Brazilian Census Bureau (Instituto Brasileiro de Geografia e Estatistica, IBGE) master sample of the Sistema Integrado de Pesquisas Domiciliares (Integrated System of Household Surveys). This is established by the census tracks of the Brazilian 2010 census, except very small samples or those that are considered special. The master sample is comprised by a collection of parts called primary sampling units (PSU). The PNS sample was selected in three stages. During the first stage, the choice of the subsample of the PSU in each section of the master sample was proportional to the size.

The second stage included randomly sampling households from the PSU selected in the first stage. One adult or 18 years or older was randomly selected from all adults in the household [23] during the last stage. The PNS questionnaire is separated into three areas. One resident responds to the first two parts and gives information on the health status as well as the household traits of all household members. The selected adult then responds to the last part and gives information on the individual questionnaire including questions on chronic conditions, oral health, lifestyle, etc. [23].

More information regarding the PNAD and PNS including datasets, survey design, and questionnaires can be found in the Brazilian Census Bureau website (www.ibge.gov.br) and in the Fundação Instituto Oswaldo Cruz (Fiocruz) (www.pns.fiocruz.br). The PNAD is conducted by the Brazilian Census Bureau and the PNS is performed by the Ministry of Health in partnership with the Brazilian Census Bureau. The PNS includes the same health status, health care access and utilization modules that are part of the PNAD health supplement in order to provide comparability across time for the health indicators in Brazil [23]. The PNS design and questionnaire followed similar protocols used by the PNAD health supplement as a way to monitor health indicators across time and regions [23]. The periodicity was also maintained as a way to facilitate the comparison of health estimates every five-years. Additional information on the study design and comparability of these studies can be found in Szwarcwald and colleagues [23]. Throughout this paper, we used PNAD and PNS de-identified public data and was regarded exempt from human subjects review.

### Participants

The 1998 PNAD interviewed 217,579 individuals ages 18 to 94 years from the 27 Brazilian states and the Federal District. In 2003, the sample was 254,714 and the 2008 PNAD interviewed 271,294 individuals. Among these respondents, 1,129 (0.5%), 1,858 (0.7%) and 1,267 (0.5%) of the participants had missing values in the covariates of interest in 1998, 2003, and 2008, respectively. The 2013 PNS interviewed 145,436 individuals 18 and older, but morbidity data are only available for the selected adult (N = 60,146). Among those with morbidity data, 7,689 (12.8%) had missing data, mostly on diabetes and hypertension status. The final sample is restricted to 52,457 with complete data on selected covariates.

#### Self-rated health

General self-rated health was obtained using the question: 'In general, would you say your health is: very good, good, fair, bad, very bad? The possible answers were grouped into two categories poor (bad, very bad) and good (fair, good and very good). Those in good health were the reference category.

#### Education

Four categorical variables for educational level (no education, primary, secondary and some college or more) were used to construct an education variable that was comparable between PNAD and PNS. No education corresponds to people who had no education or less than one year of formal schooling; ‘primary or secondary incomplete’ education are those with one to ten years of completed formal education; ‘secondary’ education are those who completed eleven years of schooling; and ‘some college or more’ are those who completed twelve or more years of schooling.

#### Other individual characteristics

A dichotomous variable for female (male as the reference group), a continuous variable for age in years; race (White as the reference category, Black, and Pardo, which included those of Asian descent and indigenous); region of residence (South, Southeast, Midwest, Northeast, North); a dichotomous variable for proxy respondent; and a dichotomous variable for private health insurance were all controlled in a regression analyses. People were classified as having health insurance if they reported having more than one health insurance. The main health problems were a previous diagnosis of depression, diabetes, heart disease, and hypertension. The questionnaire wording has altered in the past two surveys, but was similar in the 1998 and 2003 surveys.

In 1998 and 2003, one of the questions was “…have [health condition]?” (…tem [doença]?” Though, the wording changed, in 2008, to “Has a doctor or a health professional ever told that you have [health condition]” (Algum médico ou profissional de saúde disse que tem [doença]). The PNS 2013 wording is similar to the PNAD 2008 and promoted the concept of medical diagnosis “Has a doctor has given you a diagnosis of [health condition]? (Algum médico já lhe deu o diagnóstico de [doença]?). People who replied favorably per survey-year were measured as having the chronic condition and those who responded negatively as not having the negative health outcome. Pregnant women who reported being diagnosed with diabetes or hypertension were also classified as not having the chronic conditions.

### Statistical analysis

Descriptive statistics for each survey year are presented in Table 1. Table 2 presents the age-adjusted prevalence rates of poor self-rated health and prevalence ratios. Age-adjustment used the 2010 Brazilian age-distribution as standard. We calculated the absolute difference in age-adjusted prevalence rates of poor health between those with lowest and highest education. Relative educational inequalities were computed using ratios of predicted prevalence of poor self-rated health (R) in the lowest education to the predicted prevalence in the highest education group. Table 3 presents the logistic regressions for each year, adjusting for age, sex, race, region of residence, health insurance, and chronic conditions. Next, we use multivariate logistic regression to model the log-odds of reporting poor self-rated health by educational groups adjusting for age, sex, race, region of residence, health insurance, having chronic conditions, and year. Next, we include an interaction term between education and survey-year to test whether the odds of reporting poor self-rated health by educational groups differed over time. The estimations of interest are the effects of the education dummy variables as well as their interaction terms on the log odds of the outcome poor self-rated health. Both descriptive statistics and regression analyses accounted for multistage probability sampling design. Statistical analyses were performed in Stata 14.1 SE version (StataCorp, College Station, TX).

**Table 1:** Descriptive statistics, Brazil: 1998–2013.

	1998	2003	2008	2013
Poor health				
No	94.7	95.3	94.9	94.1
Yes	5.3	4.7	5.1	5.9
Education				
No education	16.1	13.6	11.8	12.9
Primary or secondary incomplete	60.9	55.8	50.1	39.4
Secondary complete	13.9	19.7	24.8	28.6
Some college or more	9.0	10.9	13.4	19.1
Mean age (SD)	39.8 (16.2)	40.2 (16.4)	41.4 (16.6)	44.1(16.8)
Sex				
Male	47.9	47.8	47.7	44.6
Female	52.1	52.2	52.3	55.4
Race				
White	56.4	53.9	50.0	49.4
Black	6.2	6.4	7.5	9.0
Pardo	37.5	39.7	42.5	41.6
Region				
North	4.4	5.3	7.3	6.7
Northeast	26.8	26.7	26.4	25.5
Midwest	7.0	7.2	7.3	7.2
Southeast	46.1	45.5	44.2	45.8
South	15.7	15.3	14.8	14.7
Health insurance				
No	73.4	73.1	71.9	67.0
Yes	26.6	26.9	28.1	33.0
Diabetes				
No	96.9	96.2	94.9	92.9
Yes	3.1	3.8	5.1	7.1
Heart				
No	94.2	94.7	94.6	95.4
Yes	5.8	5.3	5.4	4.6
Hypertension				
No	83.4	81.9	80.1	76.5
Yes	16.6	18.1	19.9	23.5
Depression				
No	92.5	94.1	94.2	91.7
Yes	7.5	5.9	5.8	8.3
Proxy respondent				
No	52.7	52.7	62.0	33.4
Yes	47.3	47.3	38.0	66.6
Sample size	216,450	252,856	270,027	52,457

**Table 2:** Age-adjusted prevalence rates of poor self-rated health, Brazil: 1998–2013.

	1998			2003			2008			2013		
	Prevalence rate	95% CI		Prevalence rate	95% CI		Prevalence rate	95% CI		Prevalence rate	95% CI	
No education	10.8	10.3	11.3	10.7	10.2	11.3	11.0	10.4	11.6	11.8	10.1	13.6
Primary or Secondary incomplete	5.1	4.9	5.2	4.7	4.5	4.8	5.3	5.1	5.4	6.1	5.6	6.6
Secondary complete	2.3	2.0	2.5	1.8	1.6	2.0	2.4	2.2	2.6	2.6	2.2	3.0
Some college or more	1.5	1.2	1.8	1.3	1.0	1.5	1.4	1.2	1.6	1.4	1.1	1.7
Total	5.7	5.5	5.9	4.9	4.8	5.1	5.0	4.9	5.1	5.2	4.9	5.5
Absolute difference	**9.3**			**9.5**			**9.6**			**10.5**		
Prevalence Ratio (R)	**7.1**			**8.4**			**7.8**			**8.5**		

Note: Absolute difference was obtained by subtracting the prevalence rate of those with some college or more from those with no education. Prevalence ratio is obtained by dividing the prevalence rate among those with no education by the prevalence rate of those with some college or more.

**Table 3:** Odds-ratios and 95% confidence intervals examining the association between educational levels and poor self-rated health, Brazil: 1998–2013.

	1998		2003		2008		2013	
VARIABLES	OR	95% CI	OR	95% CI	OR	95% CI	OR	95% CI
Education								
Primary and secondary incomplete	0.52***	0.48–0.55	0.49***	0.46–0.52	0.55***	0.52–0.58	0.59***	0.51–0.69
Secondary complete	0.24***	0.21–0.27	0.21***	0.19–0.24	0.26***	0.23–0.28	0.27***	0.21–0.34
Some college or more	0.21***	0.17–0.25	0.16***	0.13–0.19	0.18***	0.16–0.21	0.17***	0.12–0.23
Age	1.03***	1.03–1.04	1.03***	1.03–1.03	1.03***	1.02–1.03	1.02***	1.01–1.02
Female	0.99	0.95–1.04	0.92***	0.88–0.96	0.89***	0.85–0.93	1.04	0.91–1.19
Race								
Black	0.99	0.89–1.11	1.09*	0.99–1.20	1.13***	1.04–1.24	1.18	0.94–1.46
Pardo	1.11***	1.05–1.18	1.09***	1.02–1.15	1.09***	1.04–1.15	1.13	0.97–1.33
Region								
North	1.79***	1.54–2.09	1.49***	1.33–1.68	1.69***	1.51–1.89	1.88***	1.51–2.35
Northeast	1.55***	1.41–1.70	1.57***	1.44–1.71	1.52***	1.41–1.64	1.96***	1.65–2.33
Midwest	1.28***	1.14–1.44	1.29***	1.16–1.42	1.08*	0.99–1.18	1.30**	1.06–1.59
South	1.30***	1.17–1.44	1.05	0.95–1.16	1.12**	1.01–1.23	1.28**	1.03–1.57
Health insurance	0.65***	0.60–0.70	0.61***	0.57–0.66	0.61***	0.57–0.66	0.60***	0.50–0.73
Diabetes	2.20***	2.02–2.39	2.03***	1.88–2.19	2.12***	1.98–2.26	2.00***	1.67–2.39
Heart disease	2.75***	2.58–2.94	2.83***	2.65–3.03	2.65***	2.50–2.81	2.22***	1.81–2.73
Hypertension	1.76***	1.66–1.86	1.74***	1.65–1.84	1.73***	1.64–1.82	1.68***	1.46–1.93
Depression	3.93***	3.68–4.19	3.92***	3.67–4.19	4.76***	4.47–5.06	3.35***	2.77–4.04
Proxy respondent	1.00	0.96–1.05	1.08***	1.03–1.14	1.12***	1.07–1.18	1.22***	1.05–1.41
Constant	0.01***	0.01–0.01	0.01***	0.01–0.02	0.02***	0.01–0.02	0.02***	0.01–0.03
Observations	216,450		252,856		270,027		52,457	

Reference categories: no education, males, White, Southeast region, no health insurance, no diabetes, no heart disease, no hypertension, no depression and no proxy respondent.

*p<0.05.

**p<0.01.

***p<0.0001.

## Results

Table 1 provides descriptive statistics for the four survey-years. Results show that most adults in Brazil self-report having good health. The country has experienced important educational improvements during the 1998–2013 period. In particular, the proportion of people with some college or more increased from 9.0% in 1998 to 19.1% in 2013, while the proportion of those with no education declined from 16.1% in 1998 to 12.9% in 2013. The population is aging, with the proportion of adults 50 to 94 increasing over the period. The racial composition has also been changing with a higher proportion of adults self-reporting being Pardo or Black. In terms of health conditions, the prevalence rates of diabetes, hypertension, and depression has increased over time, whereas the prevalence rate of heart disease has decreased.

Table 2 provides the age-adjusted prevalence rates and prevalence ratios (R) for each year. Total prevalence of poor health among adults reached 5.7% in 1998 and slightly declined to 5.2% in 2013. There is a clear educational gradient in poor self-rated health in each year. Prevalence ratios indicate that Brazilian adults with no education have levels that are 7 to 9 times higher than those with some college or more. The difference between those with lowest and highest education increased from 1998 to 2013.

Table 3 shows the results of the logistic regressions for each year. These results focus on the associations between educational levels and poor health, controlling for additional sociodemographic and health factors. In all years, there is a clear negative gradient, in which compared to those with no education, adults with more education have lower levels of poor health. The higher the educational level, the lower the prevalence of poor health. Older age, and having chronic conditions and depression is associated with poor health. Racial differences are evident in 1998 and 2008, with minorities being more likely to self-report being in poor health than Whites. On the other hand, having health insurance is a protective factor associated with good self-rated health. There are important differences within the country, with poor health being more reported in areas outside the Southeast region, which is the most developed economically.

**Fig 1:**
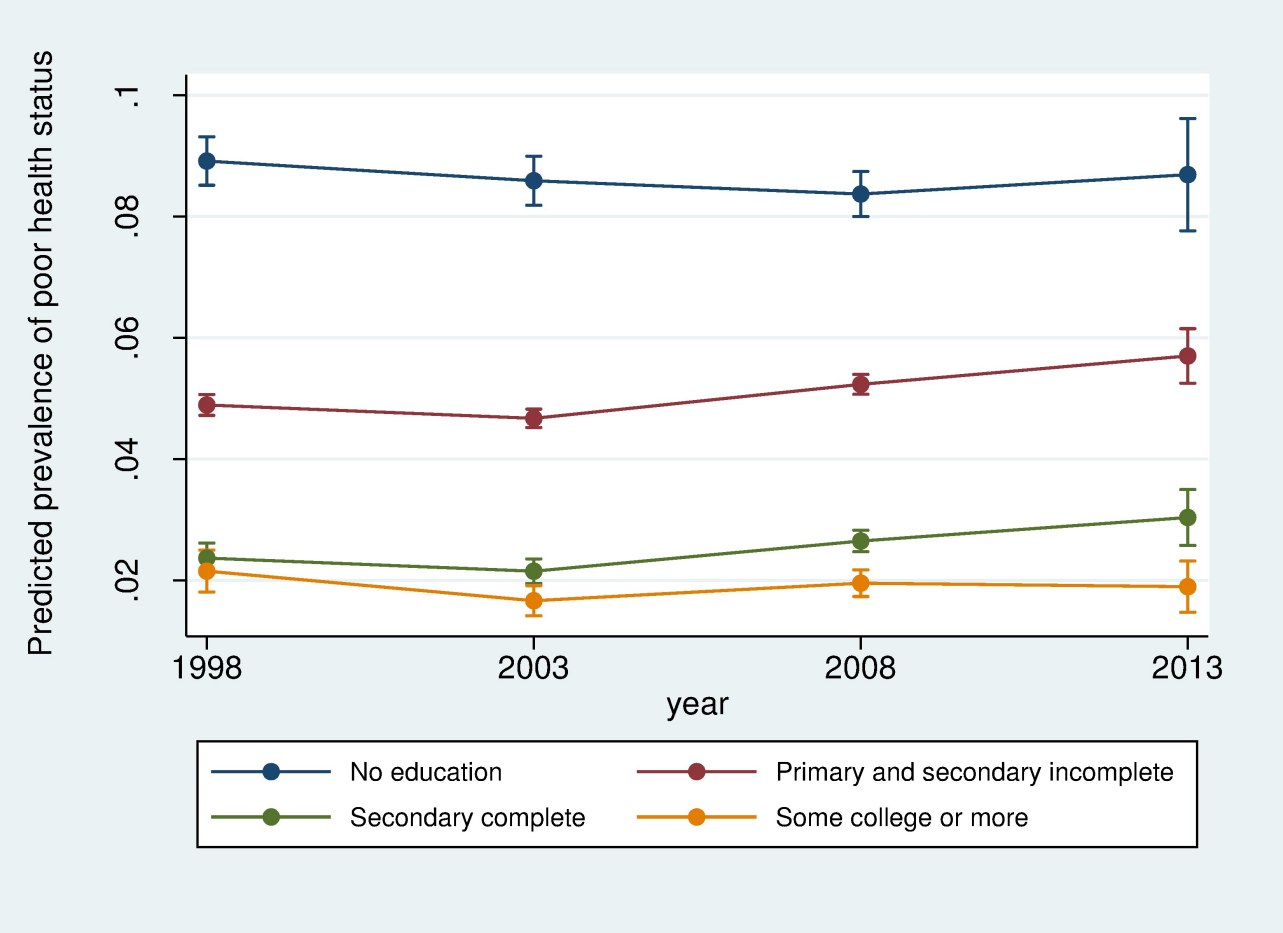
Predicted prevalence rates of poor health in Brazil by educational levels: 1998–2013.

Next, based on pooled data of all years, we examine trends in the prevalence of poor self-rated health and examine whether educational differences changed over time (Table 4). Model 1 presents the analyses including time dummies. Compared to 1998, prevalence of poor self-rated health was lower in 2003, but higher in 2013. When we add the education and time interactions in Model 2, results point out to lower prevalence in 2008 compared to 1998. In addition, compared to those with no education, there were increases in the prevalence of poor self-rated health among those with primary and secondary incomplete as well as among those with secondary complete in 2008 and 2013. On the other hand, 2003 is marked by a decrease in the prevalence among those with some college or more. Fig 1 highlights these findings based on predicted prevalence rates of poor health by educational levels for each year based on Model 2. Results for remaining variables remain similar as described in Table 3 with older age, being of racial minority, living in regions outside the Southeast, and having chronic conditions and depression being associated with poor health. On the other hand, having health insurance is a protective factor associated with better self-rated health.

**Table 4:** Odds-ratios and 95% confidence intervals examining the association between educational levels and poor self-rated health over time, Brazil: 1998–2013.

	Model 1		Model 2	
VARIABLES	OR	95% CI	OR	95% CI
Education				
Primary and secondary incomplete	0.54***	0.51–0.57	0.49***	0.46–0.52
Secondary complete	0.25***	0.23–0.28	0.22***	0.19–0.25
Some college or more	0.17***	0.15–0.20	0.20***	0.16–0.23
Year				
2003	0.94**	0.89–0.99	0.95	0.88–1.03
2008	1.02	0.97–1.07	0.92**	0.86–0.99
2013	1.11**	1.02–1.20	0.97	0.84–1.11
Education*time interactions				
Primary and secondary incomplete*1998			1.00	1.00–1.00
Primary and secondary incomplete*2003			0.99	0.92–1.07
Primary and secondary incomplete*2008			1.17***	1.08–1.26
Primary and secondary incomplete*2013			1.23***	1.05–1.45
Secondary complete*1998			1.00	1.00–1.00
Secondary complete*2003			0.94	0.80–1.11
Secondary complete*2008			1.22***	1.06–1.41
Secondary complete*2013			1.36**	1.06–1.73
Some college or more*1998			1.00	1.00–1.00
Some college or more*2003			0.79*	0.62–1.01
Some college or more*2008			0.97	0.78–1.21
Some college or more*2013			0.90	0.66–1.23
Age	1.03***	1.02–1.03	1.03***	1.02–1.03
Female	0.97	0.93–1.02	0.98	0.93–1.02
Race				
Black	1.12***	1.03–1.22	1.12***	1.03–1.22
Pardo	1.11***	1.05–1.17	1.10***	1.05–1.16
Region				
North	1.74***	1.60–1.90	1.75***	1.60–1.91
Northeast	1.67***	1.57–1.78	1.67***	1.57–1.78
Midwest	1.23***	1.14–1.32	1.23***	1.14–1.33
South	1.19***	1.10–1.28	1.19***	1.10–1.28
Health insurance	0.61***	0.57–0.65	0.61***	0.57–0.65
Diabetes	2.04***	1.90–2.19	2.04***	1.90–2.20
Heart disease	2.65***	2.50–2.81	2.65***	2.50–2.81
Hypertension	1.71***	1.63–1.80	1.71***	1.63–1.80
Depression	3.96***	3.73–4.21	3.95***	3.72–4.21
Proxy respondent	1.11***	1.06–1.16	1.11***	1.06–1.16
Constant	0.01***	0.01–0.02	0.02***	0.01–0.02
Observations	791,790		791,790	

Reference categories: no education, 1998 year, males, White, Southeast region, no health insurance, no diabetes, no heart disease, no hypertension, no depression and no proxy respondent.

*p<0.05.

**p<0.01.

***p<0.0001.

## Discussion

This study examined the educational inequalities in the prevalence of poor self-rated health in Brazil between 1998 and 2013. We found that the prevalence of poor self-rated health among adults ranged between 5–6%, which is similar to another study based on the 2006 VIGITEL, a major data collection on residents in Brazilian state capitals and the Federal District [12]. We also found that self-rated health in Brazil is marked by a strong educational gradient, with adults with lower education having worse self-rated health [12]. This study further examines whether this gradient has changed over time. Evidence shows that gap has remained significant and increased over time, with those with primary or secondary incomplete and those with secondary education experiencing worsening of self-perceived health compared to those with no education. In general, this study shows that there are important social and geographic inequalities in health in Brazil. These inequalities persist despite the expansion of the Unified Health System and programs, such as the Family Health Program and the Community Health Agents that have been shown to effectively improve health [6].

Education is an important social determinant of health in Brazil. For all years analyzed, the prevalence of poor self-rated health was higher among those with lower educational levels. This finding confirms previous studies in Brazil [12–15]. Absolute and relative measures point to large disparities between the lowest and highest educational levels. The fact that those with primary or secondary incomplete and those with secondary education experienced worsening of self-perceived health in recent years deserves attention as most Brazilians are concentrated in these educational levels. It is possible that these groups were the ones more affected by the financial crisis, as those with less education and at the bottom of social structure may have benefited with social policies, such as Bolsa Familia program (Family Allowance program) and the increase in the minimum wages [24]. Nonetheless, these results have to be taken with some caution as in analyses (not shown) that excluded those with proxy respondents, this finding was limited to 2008. Further studies should explore whether these subjective ratings were associated with worsening of objective measures of health.

Health care services in Brazil tend to be concentrated in more developed areas, particularly in the Southeast, South, and Midwest [6]. However, access to adequate health care in the North and Northeast regions lacks behind. The geographic differences are exacerbated by the concentration of private health care and insurance in the Southeast region [6]. As a result, adults residing in the North and Northeast regions in Brazil have worse perceived general health than those residing in more developed areas [12], which this study confirms. However, having access to health care is associated with better perception of general health, which is also in agreement with previous studies conducted in Brazil [19]. Efforts are needed to improve the geographic availability of services and also to address the complex mix of public and private services in all regions [6, 25].

We find that Brazilians who self-report being Black or Pardo are more likely to report having poor health. This finding corroborates with other studies that have shown that poor self-rated health is associated with racial discrimination in Brazil [26]. In fact, there is evidence that Blacks and Pardos not only have poorer socioeconomic conditions than Whites in Brazil, but they also have worse health outcomes [27, 28]. Moreover, the association between African and Native-American ancestry with poor health remains even when using genomic information [29].

Non-communicable diseases, such as hypertension, diabetes, and neuropsychiatric conditions, are the major source of morbidity and mortality in Brazil [30]. Our study confirms the negative impact of chronic conditions, such as diabetes and heart disease, on self-perceived general health of adults and older adults in Brazil [12, 17, 19, 20, 31]. Adults with depression in Brazil were four times more likely to report having poor health than those with no depression. The increased risk of poor health among those with depression confirms previous studies in Brazil with older adults [17–19]. These results highlight not only the negative impact of chronic conditions on perceived health, but also point to inequalities in health as the prevalence of these conditions is not equally distributed across social groups. In fact, chronic conditions in Brazil are more common among the poor and lower educated adults [30, 32].

As shown in another studies in Brazil, older age is associated with poor self-rated health [12, 14]. Previous studies have shown that adult and older adult women have lower levels of poor self-rated health than their male counterparts [12, 14, 33], but we do not find this association in our study. However, some of these studies did not account for differences on chronic and mental health conditions, which could explain some of the differences across studies.

A few limitations should be noted. First, our study focus on a single measure of subjective rated health. Even though this measure is widely used and has good predictive value, it has been shown that reporting of this measure can vary across social groups, including education. This discrepancy may be due to different concepts of good health across educational groups as well as differences in health expectations [34]. In fact, there is a tendency for individuals with higher educational levels to rate their health more negatively [34]. If this tendency holds in Brazil, our estimates should be seen as conservative estimates of the educational differences in health. Second, the wording of some of the questions related to health conditions changed during the period, which may have affected reporting. Both the PNS and the 2008 PNAD reinforced the concept of medical diagnosis, which differed from the previous PNAD health supplements. The accuracy of these reports may differ by educational levels as those with higher education and better access to private health insurance may be more aware of their disease status and/or better understand the medical diagnosis. Bias may also arise based on avoidance of diagnosis, which can differ across groups. In addition, self-rated health, as well as the other health conditions, were reported either by the respondent or by a proxy respondent. Previous studies in Brazil discussed the validity of the information provided by proxy respondents, particularly regarding self-reported health [35]. The use of proxy respondents is necessary in health surveys, but the validity of their reports depend on the characteristics of the proxy respondents and target person as well as on the health measure being collected [36]. There is some evidence that proxy respondents are better at reporting physical and functioning health, but less accurate in psychological measures [37]. In this study, we included a dummy variable in the regression models to assess proxy’s reporting on health. This strategy has been previously used in studies in Brazil [38, 39]. In addition, we also excluded individuals whose data had been provided by proxy respondents. Results shown in S1 Table indicate that substantive findings related to educational disparities in health are not sensitive to the inclusion of those with proxy respondents. Conclusions remain even when using LPM models rather than logistic regressions (S2 Table). However, the analyses related to time-trends in educational disparities in health differed (S3 Table). More specifically, the worsening in the health reporting among those with primary and secondary education was only found in 2008, but not in 2013.

In sum, this study points to no improvement in the general subjective health of Brazilians, even though educational levels have been increasing. In fact, data indicate worsening perceptions among those with some primary and secondary education. Health inequalities by race and region highlight the need to improve the health of socially disadvantaged groups in Brazil. Addressing chronic conditions and mental health is needed to improve self-perceptions of health in Brazil.

## Supporting information

S1 Table Odds-ratios and 95% confidence intervals examining the association between educational levels and poor self-rated health, Brazil: 1998–2013 (exclude those with proxy respondents). Reference categories: no education, 1998 year, males, White, Southeast region, no health insurance, no diabetes, no heart disease, no hypertension, and no depression.
(DOCX)

S2 Table Coefficients from LPM models and 95% confidence intervals examining the association between educational levels and poor self-rated health, Brazil: 1998–2013. Reference categories: no education, 1998 year, males, White, Southeast region, no health insurance, no diabetes, no heart disease, no hypertension, no depression and no proxy respondent.
(DOCX)

S3 Table Comparison of results using full sample and restricted sample (with no proxy respondents) using logistic and LPM models. Models further adjusted for age, sex, race, region, health insurance, medical conditions, and proxy (models with full sample). Reference categories: no education, 1998 year.
(DOCX)
