# Supplementary material for: Increasing educational inequalities in self-rated health in Brazil, 1998-2013
Source: PLoS One. 2018 Apr 30;13(4):e0196494. doi: 10.1371/journal.pone.0196494 (PMC5927445; doi:10.1371/journal.pone.0196494)
Supplement: S1 Table — Reference categories: no education, 1998 year, males, White, Southeast region, no health insurance, no diabetes, no heart disease, no hypertension, and no depression. (DOCX) [file pone.0196494.s001.docx]

**S1 Table. Odds-ratios and 95% confidence intervals examining the association between educational levels and poor self-rated health, Brazil: 1998-2013 (exclude those with proxy respondents)**

|  | 1998 | | 2003 | | 2008 | | 2013 | |
| --- | --- | --- | --- | --- | --- | --- | --- | --- |
| VARIABLES | OR | 95% CI | OR | 95% CI | OR | 95% CI | OR | 95% CI |
| Education |  |  |  |  |  |  |  |  |
| Primary and secondary incomplete | 0.59*** | 0.55 - 0.64 | 0.56*** | 0.52 - 0.60 | 0.59*** | 0.55 - 0.64 | 0.62*** | 0.46 - 0.83 |
| Secondary complete | 0.28*** | 0.23 - 0.33 | 0.23*** | 0.20 - 0.27 | 0.28*** | 0.25 - 0.31 | 0.25*** | 0.16 - 0.39 |
| Some college or more | 0.25*** | 0.20 - 0.31 | 0.16*** | 0.13 - 0.20 | 0.19*** | 0.16 - 0.22 | 0.18*** | 0.10 - 0.31 |
| Age | 1.03*** | 1.03 - 1.04 | 1.02*** | 1.02 - 1.03 | 1.02*** | 1.02 - 1.03 | 1.02*** | 1.01 - 1.03 |
| Female | 1.00 | 0.94 - 1.06 | 0.92*** | 0.87 - 0.98 | 0.92*** | 0.88 - 0.97 | 0.85 | 0.64 - 1.12 |
| Race |  |  |  |  |  |  |  |  |
| Black | 1.06 | 0.93 - 1.20 | 1.17*** | 1.05 - 1.31 | 1.22*** | 1.09 - 1.36 | 1.00 | 0.66 - 1.52 |
| Pardo | 1.18*** | 1.10 - 1.27 | 1.11*** | 1.03 - 1.19 | 1.13*** | 1.06 - 1.20 | 0.96 | 0.71 - 1.28 |
| Region |  |  |  |  |  |  |  |  |
| North | 1.85*** | 1.56 - 2.20 | 1.60*** | 1.39 - 1.83 | 1.75*** | 1.56 - 1.97 | 1.48* | 0.94 - 2.35 |
| Northeast | 1.59*** | 1.42 - 1.77 | 1.58*** | 1.43 - 1.76 | 1.59*** | 1.46 - 1.73 | 2.06*** | 1.48 - 2.85 |
| Midwest | 1.28*** | 1.12 - 1.47 | 1.23*** | 1.09 - 1.39 | 1.11** | 1.00 - 1.23 | 1.44* | 0.95 - 2.17 |
| South | 1.32*** | 1.17 - 1.49 | 1.01 | 0.90 - 1.12 | 1.15** | 1.03 - 1.27 | 1.59** | 1.08 - 2.35 |
| Health insurance | 0.65*** | 0.59 - 0.71 | 0.60*** | 0.55 - 0.66 | 0.62*** | 0.57 - 0.68 | 0.64*** | 0.46 - 0.89 |
| Diabetes | 2.17*** | 1.96 - 2.41 | 2.06*** | 1.87 - 2.26 | 2.06*** | 1.90 - 2.23 | 2.18*** | 1.54 - 3.10 |
| Heart disease | 2.81*** | 2.59 - 3.04 | 2.97*** | 2.74 - 3.21 | 2.75*** | 2.57 - 2.95 | 2.66*** | 1.77 - 4.00 |
| Hypertension | 1.79*** | 1.67 - 1.91 | 1.82*** | 1.70 - 1.95 | 1.79*** | 1.67 - 1.91 | 1.48*** | 1.13 - 1.94 |
| Depression | 3.45*** | 3.21 - 3.72 | 3.47*** | 3.21 - 3.76 | 4.43*** | 4.13 - 4.75 | 3.39*** | 2.20 - 5.21 |
| Constant | 0.01*** | 0.01 - 0.01 | 0.02*** | 0.01 - 0.02 | 0.02*** | 0.01 - 0.02 | 0.02*** | 0.01 - 0.04 |
| Observations | 113,529 |  | 133,417 |  | 168,385 |  | 13,589 |  |

Reference categories: no education, 1998 year, males, White, Southeast region, no health insurance, no diabetes, no heart disease, no hypertension, and no depression.
