# Supplementary material for: Increasing educational inequalities in self-rated health in Brazil, 1998-2013
Source: PLoS One. 2018 Apr 30;13(4):e0196494. doi: 10.1371/journal.pone.0196494 (PMC5927445; doi:10.1371/journal.pone.0196494)
Supplement: S2 Table — Reference categories: no education, 1998 year, males, White, Southeast region, no health insurance, no diabetes, no heart disease, no hypertension, no depression and no proxy respondent. (DOCX) [file pone.0196494.s002.docx]

**S2 Table. Coefficients from LPM models and 95% confidence intervals examining the association between educational levels and poor self-rated health, Brazil: 1998-2013**

|  | 1998 | | 2003 | | 2008 | | 2013 | |
| --- | --- | --- | --- | --- | --- | --- | --- | --- |
| VARIABLES | Coef | 95% CI | Coef | 95% CI | Coef | 95% CI | Coef | 95% CI |
| Education |  |  |  |  |  |  |  |  |
| Primary and secondary incomplete | -0.06*** | -0.07 - -0.06 | -0.07*** | -0.07 - -0.06 | -0.06*** | -0.07 - -0.06 | -0.06*** | -0.08 - -0.05 |
| Secondary complete | -0.07*** | -0.07 - -0.06 | -0.08*** | -0.08 - -0.07 | -0.08*** | -0.08 - -0.07 | -0.09*** | -0.11 - -0.07 |
| Some college or more | -0.07*** | -0.08 - -0.07 | -0.08*** | -0.09 - -0.07 | -0.08*** | -0.09 - -0.08 | -0.10*** | -0.11 - -0.08 |
| Age | 0.00*** | 0.00 - 0.00 | 0.00*** | 0.00 - 0.00 | 0.00*** | 0.00 - 0.00 | 0.00*** | 0.00 - 0.00 |
| Female | 0.00* | -0.00 - 0.00 | -0.00 | -0.00 - 0.00 | -0.00*** | -0.00 - -0.00 | 0.00 | -0.00 - 0.01 |
| Race |  |  |  |  |  |  |  |  |
| Black | -0.00 | -0.01 - 0.00 | 0.00 | -0.00 - 0.01 | 0.01** | 0.00 - 0.01 | 0.01 | -0.00 - 0.02 |
| Pardo | 0.00*** | 0.00 - 0.01 | 0.00** | 0.00 - 0.00 | 0.00** | 0.00 - 0.00 | 0.01* | -0.00 - 0.01 |
| Region |  |  |  |  |  |  |  |  |
| North | 0.02*** | 0.02 - 0.03 | 0.01*** | 0.01 - 0.02 | 0.02*** | 0.01 - 0.03 | 0.03*** | 0.02 - 0.04 |
| Northeast | 0.02*** | 0.01 - 0.02 | 0.02*** | 0.01 - 0.02 | 0.02*** | 0.01 - 0.02 | 0.03*** | 0.02 - 0.04 |
| Midwest | 0.01*** | 0.00 - 0.01 | 0.01*** | 0.00 - 0.01 | 0.00 | -0.00 - 0.01 | 0.01** | 0.00 - 0.02 |
| South | 0.01*** | 0.00 - 0.01 | 0.00 | -0.00 - 0.00 | 0.00** | 0.00 - 0.01 | 0.01** | 0.00 - 0.02 |
| Health insurance | -0.02*** | -0.02 - -0.01 | -0.02*** | -0.02 - -0.02 | -0.02*** | -0.02 - -0.02 | -0.02*** | -0.03 - -0.01 |
| Diabetes | 0.10*** | 0.08 - 0.11 | 0.08*** | 0.07 - 0.09 | 0.08*** | 0.08 - 0.09 | 0.07*** | 0.05 - 0.09 |
| Heart disease | 0.13*** | 0.12 - 0.14 | 0.12*** | 0.11 - 0.13 | 0.12*** | 0.11 - 0.13 | 0.08*** | 0.06 - 0.11 |
| Hypertension | 0.04*** | 0.04 - 0.04 | 0.03*** | 0.03 - 0.04 | 0.03*** | 0.03 - 0.04 | 0.03*** | 0.02 - 0.04 |
| Depression | 0.12*** | 0.11 - 0.12 | 0.11*** | 0.10 - 0.11 | 0.14*** | 0.13 - 0.14 | 0.09*** | 0.07 - 0.11 |
| Proxy respondent | 0.00*** | 0.00 - 0.01 | 0.01*** | 0.00 - 0.01 | 0.01*** | 0.01 - 0.01 | 0.01** | 0.00 - 0.01 |
| Constant | 0.01*** | 0.01 - 0.02 | 0.04*** | 0.03 - 0.05 | 0.04*** | 0.04 - 0.05 | 0.05*** | 0.03 - 0.07 |
| Observations | 216,450 |  | 252,856 |  | 270,027 |  | 52,457 |  |

Reference categories: no education, 1998 year, males, White, Southeast region, no health insurance, no diabetes, no heart disease, no hypertension, no depression and no proxy respondent.
