# Supplementary material for: Increasing educational inequalities in self-rated health in Brazil, 1998-2013
Source: PLoS One. 2018 Apr 30;13(4):e0196494. doi: 10.1371/journal.pone.0196494 (PMC5927445; doi:10.1371/journal.pone.0196494)
Supplement: S3 Table — Models further adjusted for age, sex, race, region, health insurance, medical conditions, and proxy (models with full sample). Reference categories: no education, 1998 year. (DOCX) [file pone.0196494.s003.docx]

**S3 Table. Comparison of results using full sample and restricted sample (with no proxy respondents) using logistic and LPM models**

|  | Logistic regressions | | | | LPM models | | | |
| --- | --- | --- | --- | --- | --- | --- | --- | --- |
|  | Full sample | | Restricted | | Full sample | | Restricted | |
| VARIABLES | OR | 95% CI | OR | 95% CI | Coef | 95% CI | Coef | 95% CI |
| Education |  |  |  |  |  |  |  |  |
| Primary and secondary incomplete | 0.49*** | 0.46 - 0.52 | 0.54*** | 0.51 - 0.58 | -0.07*** | -0.07 - -0.06 | -0.06*** | -0.07 - -0.06 |
| Secondary complete | 0.22*** | 0.19 - 0.25 | 0.24*** | 0.20 - 0.28 | -0.08*** | -0.08 - -0.07 | -0.08*** | -0.09 - -0.07 |
| Some college or more | 0.20*** | 0.16 - 0.23 | 0.23*** | 0.19 - 0.28 | -0.08*** | -0.08 - -0.07 | -0.08*** | -0.08 - -0.07 |
| Year |  |  |  |  |  |  |  |  |
| 2003 | 0.95 | 0.88 - 1.03 | 0.90** | 0.82 - 0.99 | -0.00 | -0.01 - 0.00 | -0.01* | -0.02 - 0.00 |
| 2008 | 0.92** | 0.86 - 0.99 | 0.88*** | 0.81 - 0.96 | -0.00 | -0.01 - 0.01 | -0.01* | -0.02 - 0.00 |
| 2013 | 0.97 | 0.84 - 1.11 | 0.86 | 0.68 - 1.08 | 0.00 | -0.01 - 0.02 | -0.02* | -0.04 - 0.00 |
| Education*time interactions |  |  |  |  |  |  |  |  |
| Primary and secondary incomplete*1998 | Ref | Ref | Ref | Ref | Ref | Ref | Ref | Ref |
| Primary and secondary incomplete*2003 | 0.99 | 0.92 - 1.07 | 1.01 | 0.92 - 1.11 | 0.00 | -0.01 - 0.01 | 0.01 | -0.00 - 0.01 |
| Primary and secondary incomplete*2008 | 1.17*** | 1.08 - 1.26 | 1.13** | 1.03 - 1.23 | 0.00 | -0.00 - 0.01 | 0.01* | -0.00 - 0.02 |
| Primary and secondary incomplete*2013 | 1.23*** | 1.05 - 1.45 | 1.21 | 0.92 - 1.60 | 0.00 | -0.01 - 0.02 | 0.02* | -0.00 - 0.04 |
| Secondary complete*1998 | Ref | Ref | Ref | Ref | Ref | Ref | Ref | Ref |
| Secondary complete*2003 | 0.94 | 0.80 - 1.11 | 0.94 | 0.76 - 1.16 | 0.00 | -0.01 - 0.01 | 0.01 | -0.00 - 0.02 |
| Secondary complete*2008 | 1.22*** | 1.06 - 1.41 | 1.21** | 1.01 - 1.46 | 0.00 | -0.01 - 0.01 | 0.01* | -0.00 - 0.02 |
| Secondary complete*2013 | 1.36** | 1.06 - 1.73 | 1.22 | 0.80 - 1.87 | -0.01 | -0.02 - 0.01 | 0.02 | -0.00 - 0.04 |
| Some college or more*1998 | Ref | Ref | Ref | Ref | Ref | Ref | Ref | Ref |
| Some college or more*2003 | 0.79* | 0.62 - 1.01 | 0.69** | 0.52 - 0.93 | -0.00 | -0.01 - 0.01 | 0.00 | -0.01 - 0.01 |
| Some college or more*2008 | 0.97 | 0.78 - 1.21 | 0.84 | 0.65 - 1.09 | -0.00 | -0.01 - 0.01 | 0.00 | -0.01 - 0.01 |
| Some college or more*2013 | 0.90 | 0.66 - 1.23 | 0.89 | 0.52 - 1.54 | -0.02* | -0.03 - 0.00 | 0.01 | -0.01 - 0.04 |

Models further adjusted for age, sex, race, region, health insurance, medical conditions, and proxy (models with full sample). Reference categories: no education, 1998 year.
